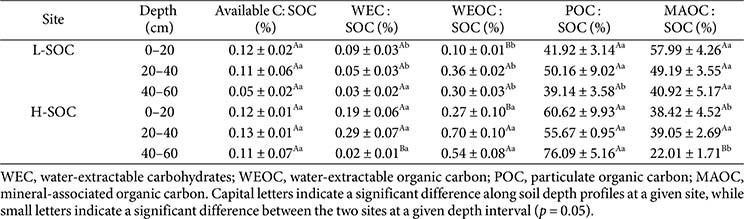# Similar Microbial Carbon Limitation with Soil Depth despite Decreasing Carbon Availability

**DOI:** 10.4014/jmb.2506.06002

**Published:** 2025-10-15

**Authors:** Seungwon Kim, Kyungjin Min

**Affiliations:** 1 Department of Agricultural Biotechnology, Seoul National University, Seoul 08826, Republic of Korea; 2 Research Institute of Agriculture and Life Sciences, Seoul National University, Seoul 08826, Republic of Korea

**Keywords:** Resource limitation, Michaelis-Menten parameters, water-extractable carbohydrate, respiration, particulate organic matter, mineral-associated organic matter

## Abstract

Carbon availability regulates the rate of microbial soil organic matter decay and respiration. However, it remains unclear how much carbon is available to microbes, and if soil organic carbon content influences microbial carbon limitation. Here, the Michaelis-Menten parameters (*V*_max_ and *k*_m_) were employed to estimate available carbon and the degree of carbon limitation along soil depth profiles (0–60 cm) at two study sites with varying soil organic carbon content. Available carbon as well as soil organic carbon decreased along soil depth profiles at both sites. Yet, the relative proportion of available carbon to the soil organic carbon did not change across soil depth intervals and sites. Overall, 0.11% (w/w) of soil organic carbon was available to microbes. In addition, the ratio of available carbon to *K_m_*, a proxy for relative carbon limitation, was invariant among soils (0.22 ± 0.03), indicating common carbon limitation. Our results highlight that the Michaelis–Menten model is useful in estimating microbial carbon availability and limitation, that microbes use only a small portion of soil organic carbon at our study sites, and that microbial communities experience similar degrees of carbon limitation regardless of soil organic carbon content.

## Introduction

Resource limitation is defined as additional growth or respiration upon resource supply [1-4]. While soil contains 20-100 times more carbon (C) than microbial biomass [5, 6], soil microorganisms often experience C limitation, as illustrated in the priming effect (*i.e.*, accelerated decomposition of soil organic C (SOC) and CO_2_ loss upon C supply) [4, 7, 8]. This suggests that not all SOC is available to microbes. Possible mechanisms for reducing microbial C availability include SOC-mineral associations and limited diffusion of microbial extracellular enzymes or substrates [9-11]. Knowledge about the magnitude of SOC that can escape from microbial decay and the fate of the supplied C can improve our understanding of the persistence of SOC. Yet, it is difficult to directly investigate how much SOC is available to microbes and what influences microbial C limitation.

**Fig. 1:**
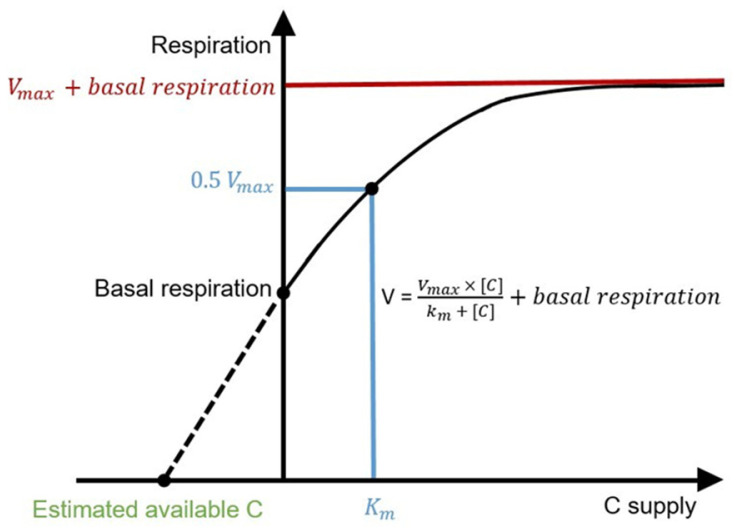
Hypothetical Michaelis-Menten graph relating microbial respiration rate as a function of C supply (V = (*V_max_* × [C]) / (*k_m_* + [C]) + basal respiration). V is microbial respiration, *V_max_* is the maximum respiration rate of extant microbes without C supply, *k_m_* is the C content required to reach 0.5 *V_max_*, and [C] is the added glucose content. The xintercept indicates the amount of C microbes use for basal respiration when there is no external C supply (here defined as available C).

The Michaelis-Menten kinetics can help us explore microbial C availability and limitation. The Michaelis-Menten kinetics were originally proposed to explain the rate of individual enzymatic reaction to substrate supply [12]. In ecology, however, the application of the Michaelis-Menten kinetics has expanded [13-16], such that microbial respiration is substituted for enzymatic reaction and substrate is replaced by one type of resource (*e.g.*, C) (Fig. 1). This approach assumes that microbial respiration is a net result of a series of enzymatic reactions, and that the supply of the rate-limiting resource, here C, can explain the overall rate of respiration [13-16]. Noticeable is that microbial respiration at no C supply (y-intercept) is not zero. This is because soil microbes respire SOC even when there is no additional C supply (basal respiration) [13]. In this case, the maximum respiration rate of extant microbes is *V_max_* plus basal respiration, not just *V_max_*. The *k_m_* is the C content required to reach 0.5 *V_max_*. Microbial respiration linearly increases with C supply at low C contents (C-limited), then gradually saturates at *V_max_* plus basal respiration as C supply increases. Once microbial respiration reaches *V_max_* plus basal respiration, additional C supply does not influence respiration (no C limitation).

If the Michaelis-Menten graph is extended and the x-intercept is calculated, the amount of SOC available to microbes to achieve basal respiration (hereafter referred to as available C) can be estimated [13]. The available C, then, refers to the amount of C that is mineralized by extant microbes during a short-term incubation [13, 15]. Because *k_m_* relates C content to *V_max_*, the ratio of available C to *k_m_* could be used to assess the degree of C limitation [14]. If the ratio is less than 1, available C is relatively less than the amount of C for the extant microbes to reach 0.5 *V_max_*. Microbes are C-limited and respiration will linearly increase with increasing C supply [13, 15]. If the ratio equals 1, microbes reach half their maximum respiratory capacity. Microbial respiration will still increase with increasing C supply up to *V_max_* plus basal respiration, but the increment in respiration upon C supply will gradually decrease [13, 15]. If the ratio is greater than 1, C is a less limiting factor. Microbial respiration will soon level off despite C supplies.

Conventionally, low C contents are thought to lead to microbial C limitation [4, 17, 18]. Previous studies reported that the lower the C content is, the smaller the microbial respiration is [19, 20]. Commonly used C contents include SOC, water-extractable OC (WEOC), or particulate organic carbon (POC) [21-23]. Especially, WEOC and POC are often considered as the indices for the potential, microbially-available C [24, 25]. While the C contents can influence the system's carrying capacity, these studies often miss direct evidence of microbial C limitation. That is, without additional respiration upon C supply, basal respiration itself does not necessarily guarantee microbial C limitation. Therefore, changes in microbial respiration with C supply need to be assessed to test the effects of C content on microbial C limitation and better understand the fate of external C.

The objectives of this study were to (1) examine how much C is available to microbes for basal respiration during a short-term incubation, (2) assess if and how much microbes are limited by C in soil, and (3) explore if SOC content influences microbial C limitation. By doing so, we hoped to understand the magnitude of SOC that can escape from microbial decay and the fate of the supplied C, a critical knowledge gap in assessing the persistence of SOC and associated soil-climate feedback. The Michaelis-Menten parameters and soils with varying SOC contents were employed for these purposes. Because SOC content decreases along soil depth profiles and varies with vegetation types [26-28], soils from spatially distinct locations were leveraged to generate a gradient of SOC contents: horizontally (under grasses vs. trees) and vertically (0–20, 20–40, and 40–60 cm) different soils.

## Materials and Methods

### Soil Collection

Soils with varying SOC contents were sampled at 0-60 cm from two study sites with relatively low and high SOC contents (hereafter referred to as L-SOC and H-SOC) (Table 1). Our preliminary data indicated that the two sites exhibited different SOC contents (L-SOC: 32.80 ± 0.86 mg C g^-1^, H-SOC: 51.10 ± 5.69 mg C g^-1^). The two study sites are located at Seoul National University, Korea (H-SOC, 37°27'26" N, 126°57'15" E; L-SOC, 37°27'31" N, 126°57'21" E). The 10-year mean annual temperature is 12.8°C, with the minimum mean temperature of -1.7°C in January and a maximum of 25.7°C in August [29]. The 10-year mean annual precipitation is 1,417.9 mm, with the minimum mean precipitation of 14.5 mm in January and a maximum of 439.1 mm in July [29]. The ambient temperature at sampling time was approximately 28.1°C. The dominant vegetation is Korean lawn grass (*Zoysia japonica* Steud) at L-SOC and pine trees (*Pinus densiflora*) at H-SOC, respectively. Both sites' soil is sandy loam (sandy skeletal, mixed, mesic family of Typic Udorthents; Dosan series) according to the Soil Information System of the Rural Development Administration [30]. Four soil cores (0–60 cm; diameter = 3.81 cm) were collected at each study site and immediately transported to the laboratory in a 4°C cooler. Soil cores were divided into three depth intervals (0–20, 20–40, and 40–60 cm) and sieved through a 2 mm sieve (total 24 soil samples = 2 sites × 4 cores × 3 soil depth intervals). The initial pH, SOC, total nitrogen, and microbial biomass C were determined on the 2 mm sieved soils (Table 1). Soil pH was determined by mixing 1 g of fresh soil with 5 mL of deionized water (S220, Mettler Toledo, Switzerland). SOC and total nitrogen concentrations were measured using the dry combustion method (Flash EA 1112, Thermo Electron Co., USA) at the National Instrumentation Center for Environmental Management. Microbial biomass C was determined using substrate-induced respiration [31].

### C Pools in Soil

Potential C pools for basal respiration—total SOC, water-extractable carbohydrates (WEC), water-extractable organic carbon (WEOC), particulate organic C (POC), and mineral-associated organic C (MAOC)—were quantified. Total SOC content was assessed on dry, 2 mm-sieved soils using the dry combustion method at the National Instrumentation Center for Environmental Management (Flash EA 1112, Thermo Fisher Scientific, USA). WEC and WEOC were extracted by mixing 10 g of fresh soil with 35 ml of 70°C deionized water, shaking the mixtures for 4 h, and centrifuging them at 3,000 ×*g* for 5 min. The supernatant was filtered through a 0.45 μm filter (Qualitative filter papers no.1, UK) and divided into two vials (one for WEC and the other for WEOC). WEC was determined following Dubois [32] using a UV spectrometer (UV/VIS Spectrophotometer, Optizen POP, Republic of Korea). Briefly, 1 ml of 5% phenol solution was added to 2 ml of the filtered solution (1:2 = phenol solution: filtered solution, *v/v*), then immediately vortexed with 5 ml of 95.5% (3:5 = mixture: sulfuric acid, *v/v*) concentrated sulfuric acid. The absorbance of the mixture was measured at 490 nm. The WEOC was analyzed at the National Instrumentation Center for Environmental Management (Sievers 5310 C, GE Analytical Instruments, USA). For POC and MAOC, the methods described in Cambardella and Elliott [33] were followed. 10 g of dry soil was dispersed with 30 mL of 0.5% sodium hexametaphosphate (1:3 = soil: liquid, w/v), and POC and MAOC were separated through wet sieving using a 53 μm sieve. The C content of each fraction was quantified using the same method employed for the SOC determination described above.

### Microbial Respiration and Michaelis-Menten Model Fitting

Microbial respiration was assessed by modifying a method by Min and Choi [34]. 20 g of fresh soil were placed in 400 ml glass jars, adjusted to 60% (w/w) water holding capacity, and pre-incubated at 24°C for 24 h to allow time for microbes to settle down after disturbance (*e.g.*, soil sampling, sieving, and water addition). Then, the jars were closed with lids equipped with non-dispersive infrared CO_2_ sensors (K30-FS, USA), and the accumulation of CO_2_ in the jars (basal respiration) was monitored. The respiration rate was calculated as μg C-CO_2_ g^-1^ dry soil h^-1^. Once finishing the quantification of basal respiration, glucose was added to the jars at 0, 0.01, 0.03, 0.05, 0.06, 0.07, 0.08, 0.1, 0.125, 0.15, 0.2, 0.3, 0.5, 0.8, 1, 2, and 4 mg C g^-1^ dry soil (408 jars = 2 study sites × 4 replicates × 3 depth intervals × 17 glucose contents). Glucose was chosen as a representative, short-term available C for microbes, because it is utilized by most microbes upon supply [35-37] and is often preferentially used over other C sources [38, 39]. Furthermore, many studies have used it to determine microbial kinetic parameters [40-42].

**Fig. 2:**
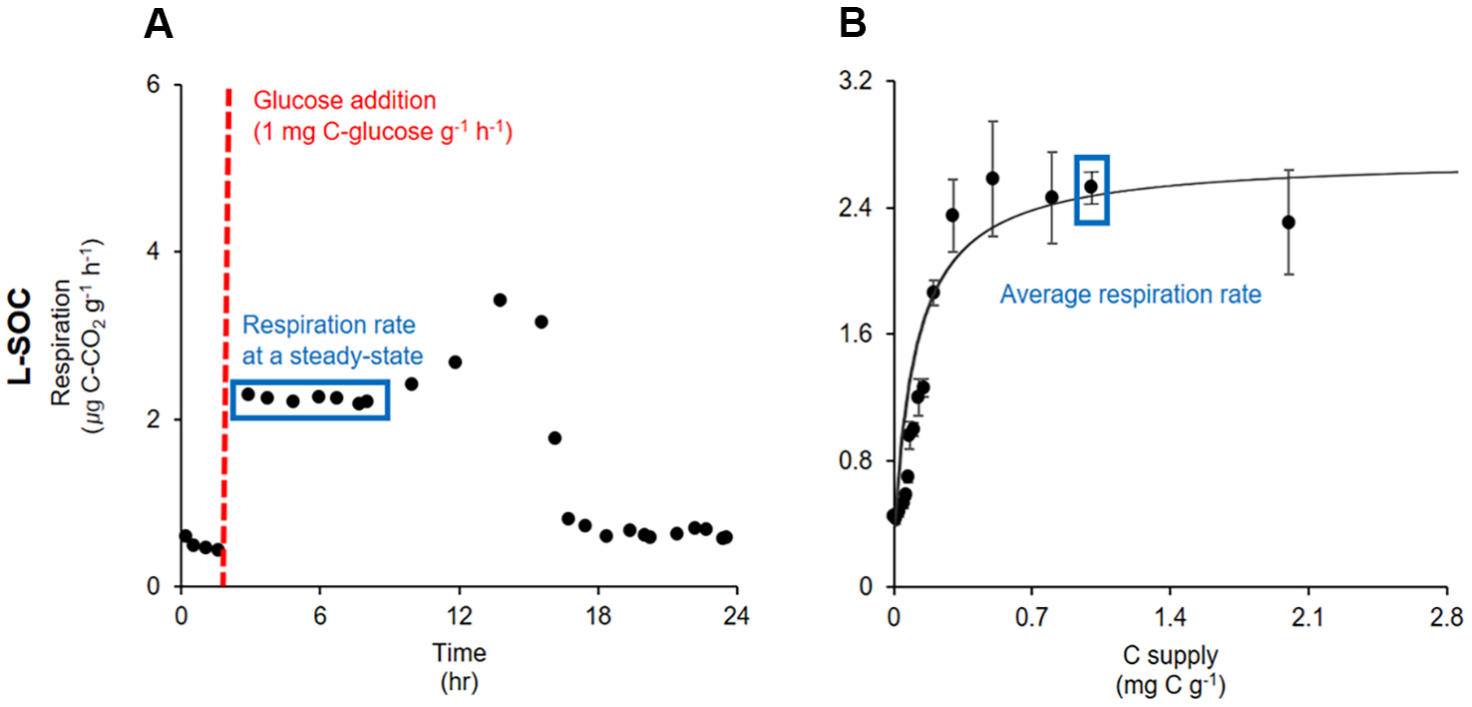
An example plot of microbial respiration from 0-20 cm soils at L-SOC (low soil organic carbon site). The red dashed line represents when glucose was added, and the blue box indicates microbial respiration at a steady state. (**B**) The average respiration rate at a steady state was plotted against C supply, and the Michaelis-Menten kinetics was fitted to the data.

**Fig. 3:**
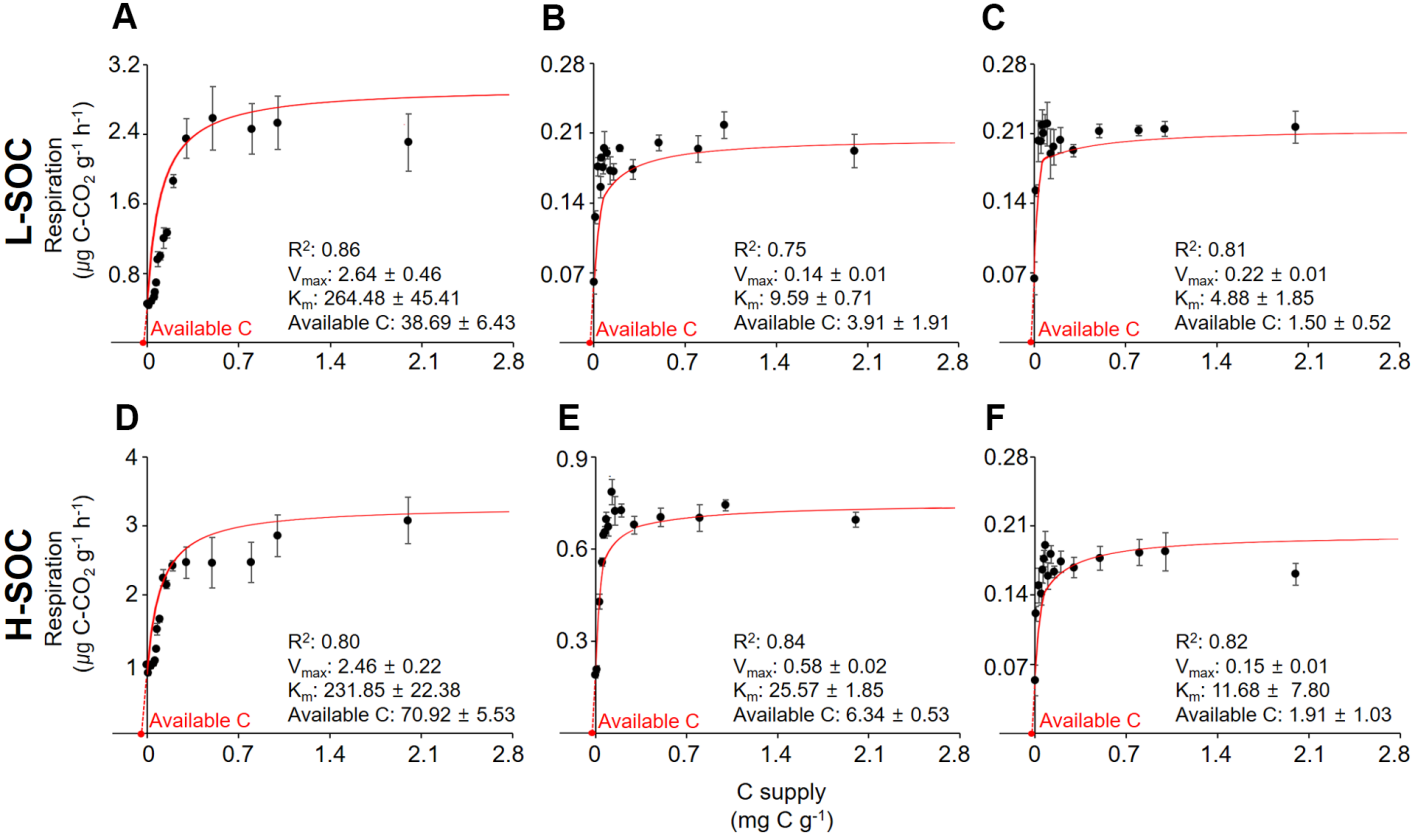
The Michaelis-Menten graphs and parameters at each site (L-SOC, A-C; H-SOC, D-F) and depth interval (0–20 cm, A and D; 20–40 cm, B and E; 40–60 cm, C and F).

As soon as glucose was added, microbial respiration increased and maintained for approximately 4 h (Fig. 2A). This relatively stable respiration is considered to result from active microbes utilizing the added C without growth [43, 44]. Please note that initial microbial communities exponentially grew and continued to respire even after the steady state (Fig. 2A). Microbial respiration was recorded at a steady state and plotted against the C supply to assess the initial microbial responses to the C supply (Fig. 2B). Then, the Michaelis-Menten model was fitted to the data using the *nls* function in the *stats* package in R, *V_max_* and *k_m_* were calculated, and available C was estimated using equation 1 (Fig. 3).

$$V=\frac{V_{\max}\times \left[C\right]}{k_{m}+\left[C\right]}+\text{basal respiration}$$ (1)

, where *V_max_* is the maximal respiration rate without any glucose or SOC, [C] is the content of added glucose, *k_m_* is the half-saturation constant, and basal respiration is microbial respiration at a glucose content of 0. The available C in the soil was estimated by solving the equation when V is equal to 0.

### Statistical Analyses

**Fig. 4:**
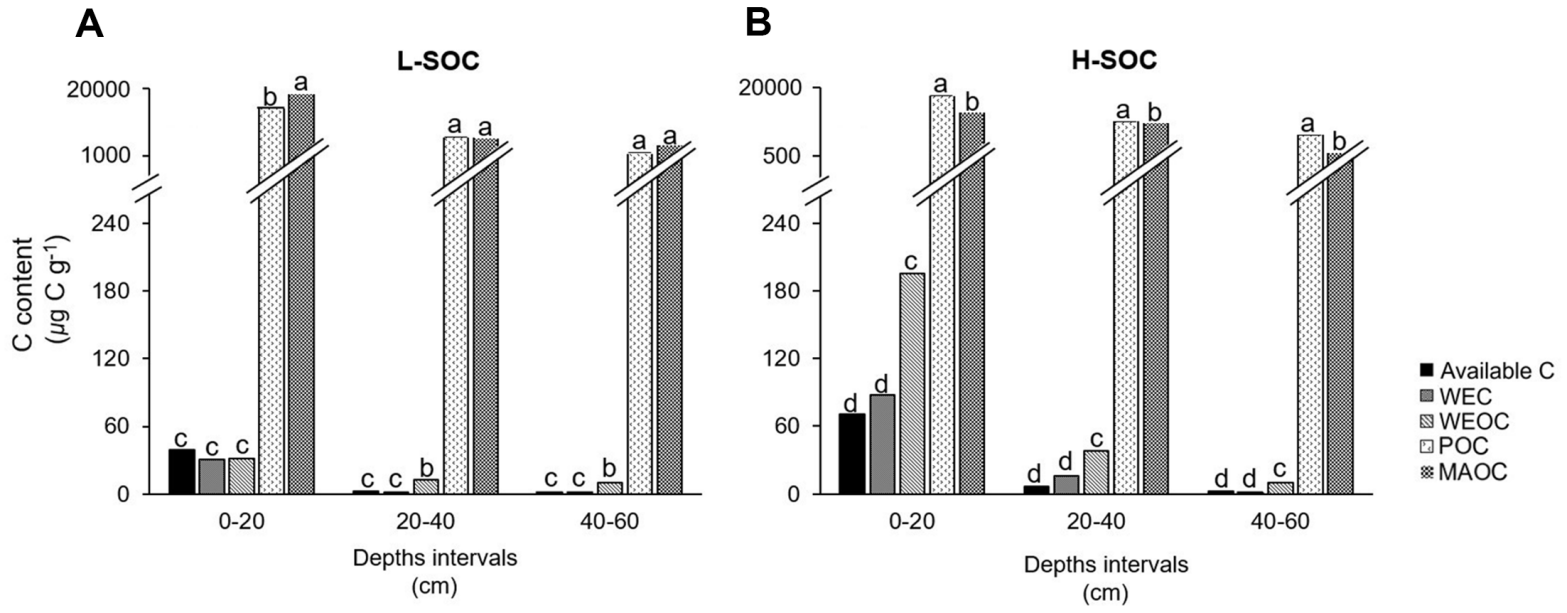
Available C and soil C pools at L-SOC (A) and H-SOC (B) sites along soil depth profiles (0–20, 20–40, and 40–60 cm). Soil C pools were directly quantified, whereas available C was estimated using the Michaelis-Menten parameters and the equation (1) (see Fig. 1).

**Fig. 5:**
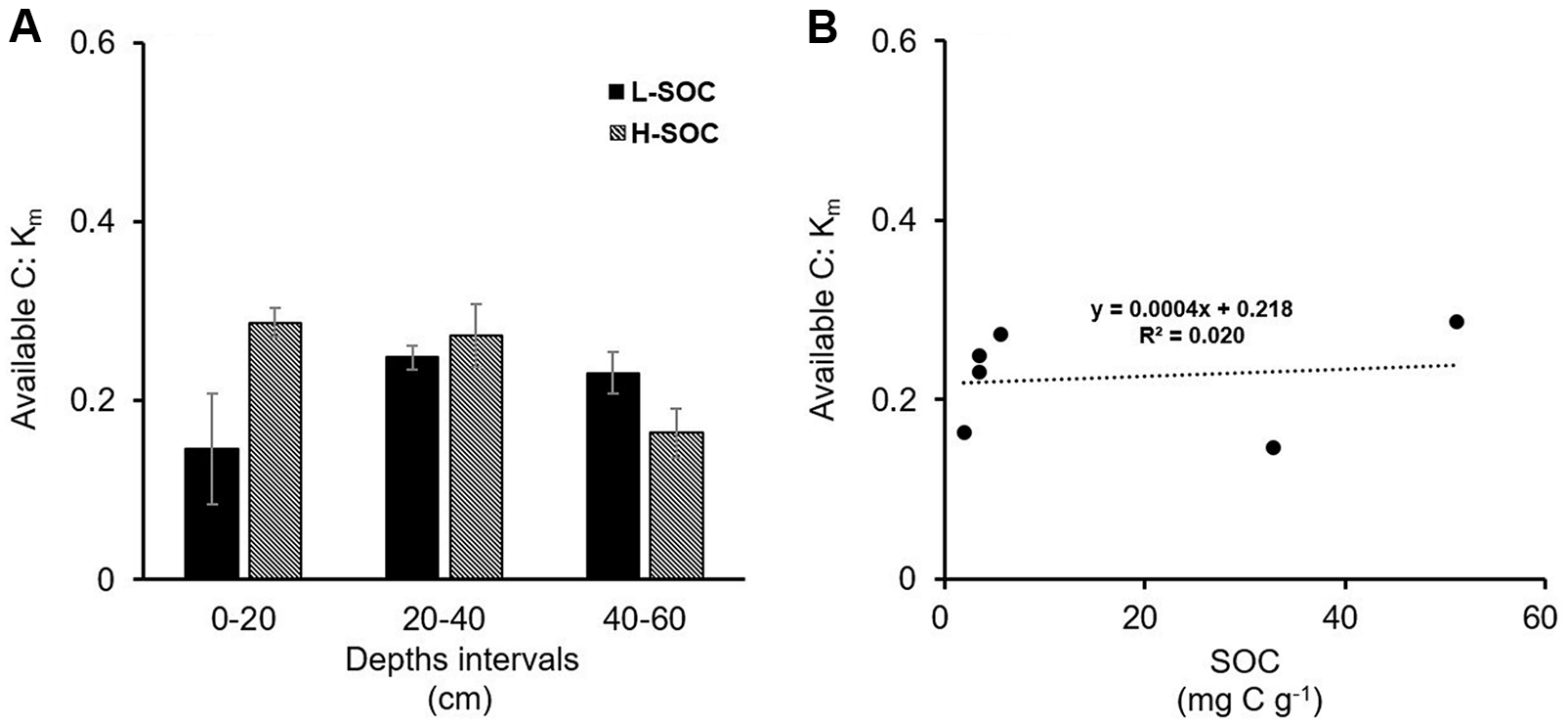
(A) The ratio of available C to *k_m_* at each site and depth interval. (B) The relationship between the available C: *k_m_* ratio and total SOC content.

Before statistical analysis, the data were checked for normal distribution. If not, data were log-transformed to meet the normality. Then, a two-way ANOVA was performed using soil depth intervals and study sites as independent variables, and available C, pH, SOC, TN, microbial biomass, and the relative proportions of available C, WEC, WEOC, POC, and MAOC to SOC as dependent variables (aov function) (Tables 1 and 2). At a given depth interval and study site, available C, WEOC, WEC, POC, MAOC, and the available C to *k_m_* ratio were compared with one another using one-way ANOVA (aov function) (Figs. 4 and 5). If a significant difference was observed (*p* < 0.05), a post hoc test was conducted (Tukey HSD function). Linear regression analysis was used to examine the relationship between SOC content and the available C to *k_m_* ratio. All statistical analyses were performed in R (R Core Team 4.3.1, 2024).

## Results and Discussion

Short-term available C was estimated using the Michaelis-Menten parameters (Fig. 3), and compared to the soil C pools with varying turnover time (Fig. 4). Overall, available C decreased along soil depth profiles, ranging from 70.92 ± 5.53 μg C g^-1^ at 0–20 cm and to 1.50 ± 0.52 μg C g^-1^ at 40–60 cm (Fig. 3). For a given depth interval, available C was higher at H-SOC than at L- SOC. A significant positive relationship was found between available C and total SOC (available C (μg C g^-1^) = 1.193 × total SOC (mg C g^-1^) – 1.161, R^2^ = 0.998).

Estimated available C was remarkably similar to WEC at all depth intervals and sites (Fig. 4). In contrast, available C was always lower than POC and MAOC for a given depth interval and site (*p* < 0.001 for all). While WEOC was similar to available C at 0–20 cm and in the L-SOC, it was greater than available C at other depth intervals. This implies that WEOC may overestimate microbially available C. The diversity of chemical compounds in WEOC may explain this discrepancy. WEOC contains diverse compounds with varying turnover times [45, 46]. For example, amino acids and simple sugars can be easily assimilated and respired by microbes, while phenolics can reside in soil for a relatively long period. When the Michaelis-Menten approach was applied to the respiration–C supply data reported in the literature, available C was estimated to be comparable to that in our study. For example, available C was 3.03 μg C g^-1^ ± 0.04 at 0–20 cm in a deciduous forest [15], 10.62 μg C g^-1^ at A-horizon in agricultural land [44], 22.19 ± 1.42 μg C g^-1^ in a grassland [37], and 100.43 ± 14.84 μg C g^-1^ at 0–10 cm in a spruce-dominated forest [47].

Next, the available C, WEC, WEOC, POC, and MAOC were corrected for the SOC content to check the relative proportion of each C pool to the total C in the soil (Table 2). While available C and SOC varied with depth profiles and sites (Figs. 3 and 4, Table 1), the relative proportion of available C to SOC was invariant (Table 2). On average, the available C to SOC ratio was 0.11% (w/w), indicating that most SOC (99.89%) was unavailable to microbes during the short-term incubation and escaped from microbial decay at our study sites. Our estimate is consistent with the values from the literature: The available C to SOC was estimated at 0.01 ± 0.002% (w/w) in a deciduous forest [15], 0.04 ± 0.003% (w/w) in agricultural land [44], 0.02 ± 0.001% (w/w) in a grassland [37], and 0.06 ± 0.01% (w/w) at 0–10 cm in a spruce-dominated forest [47].

The protection of SOC may have reduced microbial C availability. When SOC is bound to the mineral surface, it can avoid microbial decay and associated respiratory loss [48-50]. However, the POC and MAOC contents in this study indicated that the physical protection of SOC alone was insufficient to explain the reductions in C availability. For example, MAOC only represented 22.01–57.99% (w/w) of SOC (Fig. 4, Table 2), being smaller than the unavailable C (99.89% of the SOC). Also, POC, the presumably available form of SOC relative to MAOC, was still 200–1,000 times more abundant than the estimated available C (Fig. 4, Table 2). While our estimation of available C was based on the short-time incubation and thus likely to be lower than the actual available C in soil, the substantial difference between POC and available C suggests that not all POC was accessible to microbes.

Two scenarios can be put forward to explain the lower available C than POC. First, dissolution and diffusion may have played a more important role in reducing C availability than SOC protection in this study. Regression analyses reveal that WEC was the best predictor of available C (available C = 0.85 × WEC + 0.51, R^2^ = 0.97, *p* < 0.001) and basal respiration (basal respiratio*n* = 0.011 × WEC + 0.07, R^2^ = 0.97, *p* < 0.001). This implies that dissolved carbohydrates are the primary source of microbial respiration. Despite the importance of fueling respiration, however, the production of dissolved carbohydrates can be low in soils. For instance, the WEC represented only 0.02–0.29% (w/w) of the total SOC in this study (Table 2). The limited interaction of high molecular carbohydrates and extracellular enzymes that break down those molecules can reduce the production of dissolved carbohydrates [51, 52]. Cellulose and hemicellulose, the two most representative high molecular carbohydrates in soil, are large (> 100 μm) and insoluble in water [53, 54]. As such, the transport of these molecules largely depends on discrete, random events (*e.g.*, rain, earthworm movement), decreasing the likelihood of enzyme-substrate interaction.

Second, heterogeneity in the inherent biochemical recalcitrance among POC components could have decreased the decomposability of POC. POC is operationally defined as sand-sized organic debris [33, 55], which typically forms via physical fragmentation of the structural plant parts (*e.g.* lignin) [55, 56] and aggregate formation [57, 58]. Common components of POC include cellulose, lignin, and plant-derived lipids [59-61]. Lehmann [62] argued that high molecular diversity could decrease the rate at which molecules are decomposed by microbes, because the energy return on the investment in producing a suite of extracellular enzymes is relatively small.

Next, the available C to *k_m_* ratio was calculated to assess if and how much microbes are limited by C (Fig. 5). The ratio of available C to *k_m_* was always less than 1, pointing to microbial C limitation (Fig. 5A). In addition, the available C: *k_m_* ratio was invariant along a gradient of SOC contents, with an average of 0.22 ± 0.03 (Fig. 5B). This implies that higher SOC simultaneously increased available C and *K_m_*. Indeed, SOC significantly influenced available C (available C (μg C g^-1^) = 13.74 × SOC (mg C g^-1^) - 1.98, R^2^ = 0.99) and *k_m_* (km (μg C g^-1^) = 55.10 × SOC (mg C g^-1^) + 1.01, R^2^ = 0.87) at our study sites. Microbes in soils with higher SOC contents likely generated less efficient enzymes (higher km), leading to a similar ratio of available C to *k_m_* regardless of SOC contents. Together, our results demonstrate that microbial C limitation is common across a gradient of SOC contents at our study sites, and that SOC contents have little impact on the degree of C limitation. If our observations are applicable to soils in general, microbial respiration will increase with external C supply. Consistent with our prediction, fresh C stimulates microbial decay of SOC and respiration in deep soil layers with low [63, 64]. Likewise, the maximum growth rate upon C supply, which was estimated from microbial respiration, was similar between the surface and deep soil microbes, despite the differences in the SOC contents [65]. If climate change increases plant-derived C inputs into soil, SOC stock may not increase. Instead, microbial respiration is likely to be stimulated, providing a positive feedback to climate.

Some limitations of this study are acknowledged. First, glucose is not the only C that microbes use in soils. Microbes can quickly take up simple sugars, amino acids, and organic acids [66-68]. However, many studies supply glucose to soil for assessing microbial respiration [69, 70], because most microbes use glucose over other organic compounds. In addition, our results of similar contents between the estimated available C and the quantified WEC (Fig. 4), and WEC being the best predictor of available C and basal respiration (check above) suggest that our approach is still suitable to explore microbial C availability in soils. Future studies that explore the effects of different C sources on the available C estimation will improve our understanding of microbial C limitation in soils.

Second, available C may have been underestimated due to the relatively short-term incubation (hours; Fig. 2). The turnover time of SOC pools ranges from months to years and millennia [71, 72]. Hence, hours of incubation may be too short for us to accurately estimate available C. Indeed, our estimation of available C was lower than WEC and WEOC, the two commonly used indicators for readily accessible C in literature (Fig. 4). Despite the underestimation of available C, however, there was evidence that glucose was readily consumed by microbes (Fig. S1). Glucose recovery once microbial respiration returned to its basal respiration was 9.00 ± 3.22% (w/w) at 0-20 cm, 26.94 ± 2.14% (w/w) at 20-40 cm, and 42.70 ± 6.70% (w/w) at 40-60 cm. Given that glucose recovery just upon the addition was > 95.63 ± 2.46% (w/w), the relatively low glucose recovery, especially at 0-20 cm, was due to microbial consumption of glucose, not low glucose extraction efficiency. At deeper soil layers, low microbial biomass likely led to the relatively high glucose recovery. These results imply that microbes consumed a substantial amount of added glucose even during the short-term incubation in this study.

Third, our soil samples represent neither a broad range of SOC contents (only 0.19–5.1%, w/w) nor diverse ecosystems (just one grassland and one forest). If more diverse soil samples had been obtained, a more generalizable conclusion could have been reached. However, when the Michaelis-Menten approach was applied to the respiration-C supply data reported in the literature, similar results were obtained across forest, agriculture, and grassland soils with diverse SOC contents [15, 37, 44, 47]. This suggests that our observations of low microbial C availability and common C limitation are still valid and agree with other studies.

## Conclusion

This study highlights that microbes have access to only a small proportion of SOC during short-term incubation, and that similar degrees of C limitation occur in soils with varying SOC contents. Our study shows that most SOC escapes from microbial utilization either due to physical protection or limited diffusion. Given the importance of C availability in regulating microbial respiration, more investigation into the formation, content, and composition of available C would improve our assessment and prediction of the persistence of SOC under changing environmental conditions.

## Supplemental Materials

Supplementary data for this paper are available on-line only at http://jmb.or.kr.



## Figures and Tables

**Table 1:** Initial soil characteristics.

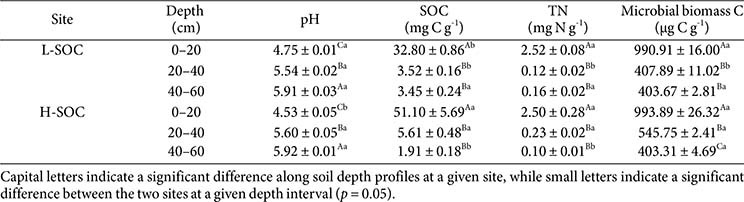

**Table 2:** Relative abundance of available C, WEC, WEOC, POC, and MAOC to total SOC contents.