# Supplementary material for: Similar Microbial Carbon Limitation with Soil Depth despite Decreasing Carbon Availability
Source: J Microbiol Biotechnol. 2025 Oct 15;35:e2506002. doi: 10.4014/jmb.2506.06002 (PMC12549229; doi:10.4014/jmb.2506.06002)
Supplement: Supplementary file 1 [file jmb-35-e2506002-supple.pdf]

J. Microbiol. Biotechnol. 2025. 35: e2506002

https://doi.org/10.4014/jmb.2506.06002

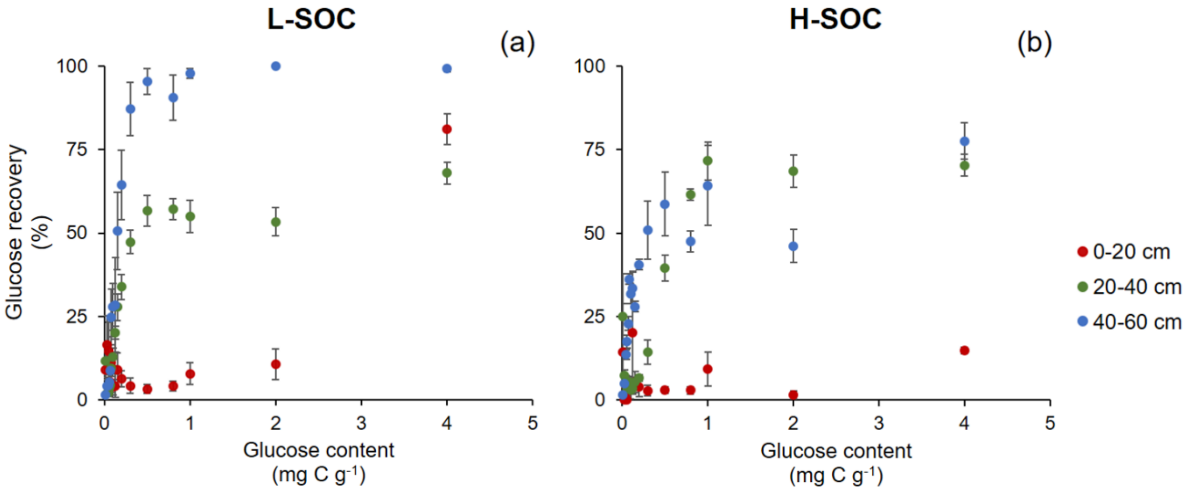

Fig. S1. Percent glucose recovery along soil depth profiles (0-20, 20-40, 40-60 cm) at L-SOC (a) and H-SOC (b) once microbial respiration returned to basal respiration.
